# Two Arabidopsis promoters drive seed-coat specific gene expression in pennycress and camelina

**DOI:** 10.1186/s13007-023-01114-x

**Published:** 2023-12-06

**Authors:** Xin Li, Victoria Yell, Xu Li

**Affiliations:** 1 Department of Plant and Microbial Biology, North Carolina State University, Raleigh, NC 27695 USA; 2 Plants for Human Health Institute, North Carolina State University, Kannapolis, NC 28081 USA

**Keywords:** Seed coat, Promoter, Pennycress, Camelina, Biofuel

## Abstract

**Background:**

Pennycress and camelina are two important novel biofuel oilseed crop species. Their seeds contain high content of oil that can be easily converted into biodiesel or jet fuel, while the left-over materials are usually made into press cake meals for feeding livestock. Therefore, the ability to manipulate the seed coat encapsulating the oil- and protein-rich embryos is critical for improving seed oil production and press cake quality.

**Results:**

Here, we tested the promoter activity of two Arabidopsis seed coat genes, *AtTT10* and *AtDP1*, in pennycress and camelina by using eGFP and GUS reporters. Overall, both promoters show high levels of activities in the seed coat in these two biofuel crops, with very low or no expression in other tissues. Importantly, *AtTT10* promoter activity in camelina shows differences from that in Arabidopsis, which highlights that the behavior of an exogenous promoter in closely related species cannot be assumed the same and still requires experimental determination.

**Conclusion:**

Our work demonstrates that *AtTT10* and *AtDP1* promoters are suitable for driving gene expression in the outer integument of the seed coat in pennycress and camelina.

**Supplementary Information:**

The online version contains supplementary material available at 10.1186/s13007-023-01114-x.

## Background

To mitigate future reliance on fossil fuels, there has been a rising interest in the development of non-food oilseed crops such as pennycress (*Thlapsi arvense*) and camelina (*Camelina sativa*) for biofuel production. Both pennycress and camelina produce seeds with more than 30% dry weight as oil, and the seed oil fatty acid profiles allows for easy conversion to biofuels which meet the U.S. Renewable Fuels Standard and can be used as sustainable aviation fuel [1–5]. They require low agricultural input such as water, pesticides, and fertilizer, and perform well on marginal lands [6, 7]. Moreover, pennycress and camelina are cold tolerant and have a short life cycle, allowing them to be planted as a fallow replacement crop in double- or relay-cropping systems [8–14]. In addition to increasing the land use efficiency and productivity per unit area, camelina and pennycress also serve as winter cover crops that provide many environmental benefits, including limiting soil erosion and nutrient loss, improving water quality, suppressing weed growth, and increasing pollinator abundance [13, 15–18].

The seed coat is a maternal tissue that derives from the outer and inner integuments of the ovule during seed embryogenesis [19–21]. It not only serves as a physical barrier that protects the embryo, but also allows the embryo to sense environmental cues [22]. After double fertilization, the embryo and endosperm start to develop, and the seed coat also undergoes a series of developmental changes. During the early stages (globular to linear cotyledon stage) of Arabidopsis seed development, the inner integument of the seed coat comprises three layers of cells and the outer integument is composed of two cell layers [20, 23]. When the embryo reaches the green mature stage, the outside cell layer of the outer integument develops mucilage and the inner cell layer experiences significant cell wall thickening [20], while the inner integument accumulates proanthocyanidins [24]. Also, an endosperm layer is tightly attached to the inside of the inner integument. During the transition from the linear cotyledon stage to the green mature stage, the cotyledons also expand greatly in size and storage reserves (oil, proteins, etc.) start to accumulate. After the green mature stage, seeds enter a brown mature stage and start to lose water, eventually becoming dormant and dry [21]. Although other *Brassicaceae* species may differ from Arabidopsis in the number of cell layers contained in outer and inner integuments during development, they all have the same overall seed coat structure layout [20, 25].

Promoters are generally considered to be the major determinant in controlling a gene’s temporal and spatial expression patterns, and are used as such in synthetic biology research and applications [26]. Several seed coat-specific genes have been experimentally identified in the model plant *Arabidopsis thaliana* [23, 27–32]. The promoter of some of these genes were shown to be able to drive gene expression in canola (*Brassica napus*) in a seed coat-specific manner [33–35]. Like canola, pennycress and camelina are members of the *Brassicaceae* family and are closely related to Arabidopsis [36]. Because of this similarity, it is expected that Arabidopsis promoters may also be used for seed coat-specific expression in these two oilseed crops.

Here we report the testing of the promoters of two Arabidopsis genes, *AtTT10* (*At5g48100*) and *AtDP1* (*At4g11180*), in pennycress and camelina. Using both the GUS and eGFP reporters, we found that the predominant activities of *AtTT10* and *AtDP1* promoters occur in the outer integument of seed coat, demonstrating that they can be used for targeted modification of seed coat traits in these two emerging biofuel oilseed crops.

## Materials and methods

### Plant material and growth condition

Pennycress spring annual line ‘MN108’ [37] and camelina cultivar ‘Suneson’ (Montana State University releases) were grown in environment controlled growth chambers under 16 h light/ 8 h dark periods (light intensity 150 µmol m^–2^ s^–1^) with 50% humidity at 21 °C. Two to four seeds were sown into a 6-inch pot with soil mix “Sunshine” (Sun Gro Horticulture, Agawam, MA) supplemented with Scotts Osmocote Plus slow release fertilizer (Hummert International, Earth City, MO, USA).

### Generation of promoter-reporter constructs

A 2 kb promoter upstream of *AtTT10* (*At5g48100*) gene coding region and a 1.2 kb promoter upstream of *AtDP1* (*At4g11180*) gene coding region were PCR amplified from Arabidopsis Col-0 with primers oXL1968/ oXL1969 and oXL1970/ oXL1971, respectively (supplemental Table S1). They were fused with an eGFP (enhanced green fluorescent protein)-GUS (β-glucuronidase) dual reporter gene, and an HSP terminator sequence into pEarleyGate103 binary vector linearized with *Ase*I and *Pvu*I, using NEBuilder® HiFi DNA Assembly (New England Biolabs, Ipswich, MA, USA, Cat. No. E2621), forming the final constructs *AtTT10p:eGFP-GUS* and *AtDP1p:eGFP-GUS* (supplemental Fig. S1).

### *Agrobacterium*-mediated transformation of pennycress and camelina via floral dip

A modified floral dip method [38–40] was used to transform pennycress and camelina. Five weeks old camelina plants and six weeks old pennycress plants were used for transformation. Single colonies of *Agrobacterium tumefaciens* strain GV3101 transformed by electroporation with binary vectors were picked into 5 mL LB media with proper antibiotic selection, and after overnight shaking incubation at 30 °C, transferred to 500 mL LB media with proper antibiotics and shake incubated overnight at 30 °C. The agrobacteria cells were spun down for 20 min at 4,000 rpm and the cell pellets were resuspended in 500 mL infiltration media (5% sucrose and 0.05% (v/v) silwet-77). Pennycress and camelina floral buds were dipped into the infiltration media with agrobacteria and treated with vacuum at -0.9 to -1 bar for 5–10 min. The inflorescences were then wrapped with plastic film and the plants were laid down and kept in the dark in the growth chamber to recover overnight, before returning to the normal growth condition the next morning.

### Identification of transgenic pennycress and camelina

Seeds were surface sterilized with sterilization solution (20% bleach and 0.02% Tween-20 in distilled deionized water) for 15 min with constant shaking at room temperature, and then washed with distilled deionized water for five times. The surface sterilized seeds were then dispensed to MS (Murashige and Skoog) solid media containing hygromycin (MS salt 4.33 g L^− 1^, sucrose 1% (w/v), agar 0.7% (w/v), pH adjusted to 5.7, hygromycin 30 µg mL^− 1^). Plates were then cold treated in darkness at 4 °C for 48 h for seed stratification, before being moved to a growth chamber. Hygromycin resistant seedlings (supplemental Fig. S2) were transferred to soil and grown to full maturity, and transgene presence was confirmed by PCR (supplemental Table S1).

### Histochemical GUS staining

Pennycress and camelina seeds, one week old seedlings grown on MS plates, rosette leaves from mature plants, inflorescence, and silicles (seeds removed) were collected into ice-cold PBS buffer (137 mM NaCl, 2.7 mM KCl, 10 mM Na_2_HPO_4_, 1.8 mM KH_2_PO_4_, pH 7.4) before GUS staining. Seeds at appropriate developmental stages were quickly hand-dissected along the longitudinal axis, and the embryo, inner integument with attached peripheral endosperm, and outer integument layers were collected separately. Immediately after harvesting, PBS buffer was completely removed, and GUS staining solution (14 mM NaH_2_PO_4_, 36 mM Na_2_HPO_4_, 2 mM K_4_[Fe(CN)_6_], 2 mM K_3_[Fe(CN)_6_], 0.2% Triton X-100, 10 mM EDTA, 2 mM X-GLUC (5-Bromo-4-Chloro-3-Indoyl-Beta-D-Glucuronide, Gold Biotechnology, St. Louis, MO, USA, Catalog number G1281)) was added to each sample. Samples were placed under vacuum at -1 bar for 10–15 min, and then incubated at 37 °C for 2 h. Afterwards, the GUS staining solution was completely removed, and the samples were washed with 70% ethanol at 4 °C until the tissue was fully cleared (about 30–60 min for developing seeds, 2 days for seedlings and silicles, and 5–7 days for mature leaves and floral tissue). When necessary, stained and cleared samples were mounted to slides for microscopic analysis using a dissecting fluorescence microscope (Zeiss SteREO Discovery.V20) or an upright fluorescence microscope (Zeiss Model Axio Imager M1) with 50% glycerol. Microscopic images were taken with a Zeiss AxioCam ICc5 camera and processed with Adobe Photoshop and Illustrator software.

## Results

### Generation of *AtTT10* and *AtDP1* promoter-reporter lines in pennycress and camelina

*AtTT10* (also known as *AtLAC15*) and *AtDP1* promoters were amplified from Arabidopsis Col-0 genomic DNA and assembled with an eGFP-GUS dual reporter into a binary vector (supplemental Figure S1). The resulting constructs were introduced into pennycress spring annual variety ‘MN108’ and camelina cultivar ‘Suneson’ by *Agrobacterium*-mediated transformation (see materials and methods). Transgenic T_1_ generation plants were selected by hygromycin resistance on MS medium (supplemental Figure S2) and the presence of the transgene was confirmed by PCR (supplemental Table S1).

### The activities of *AtTT10* and *AtDP1* promoters in pennycress and camelina are mainly detected in seed coat

**Fig. 1:**
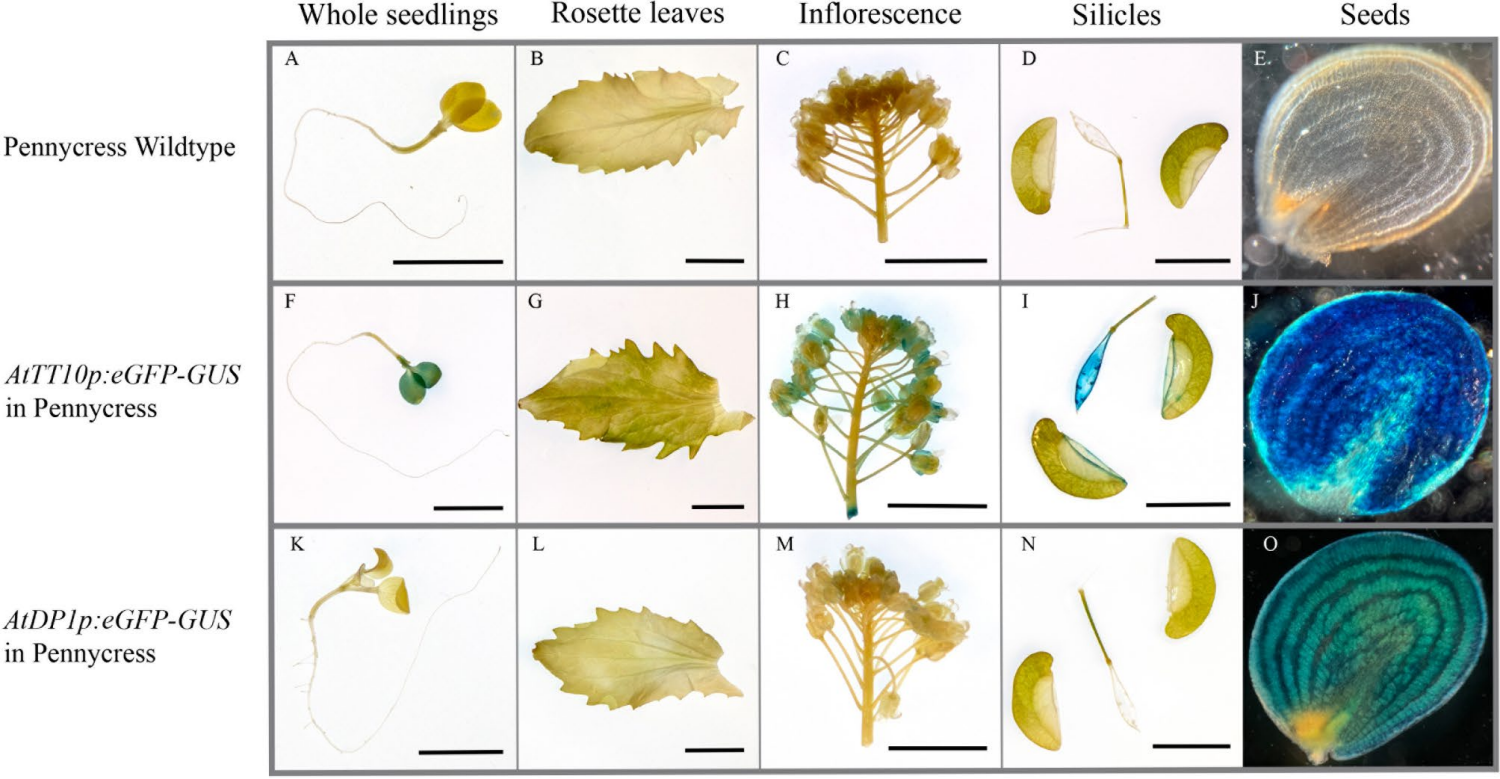
GUS signals showing *AtTT10* and *AtDP1* promoters activities in transgenic pennycress different tissues. One week old whole seedlings (**A**, **F** and **K**), rosette leaves of mature plants (**B**, **G** and **L**), inflorescence (**C**, **H** and **M**), silicles (hand peeled open and removed seeds) (**D**, **I** and **N**) and seeds (cut open along the longitudinal direction with the cutting sides facing down) (**E**, **J** and **O**) of wildtype (**A-E**), transgenic line carrying *AtTT10p:eGFP-GUS* (**F-J**) and transgenic line carrying *AtDP1p:eGFP-GUS* (**K-O**) were treated with GUS staining. Scale bars = 1 cm

**Fig. 2:**
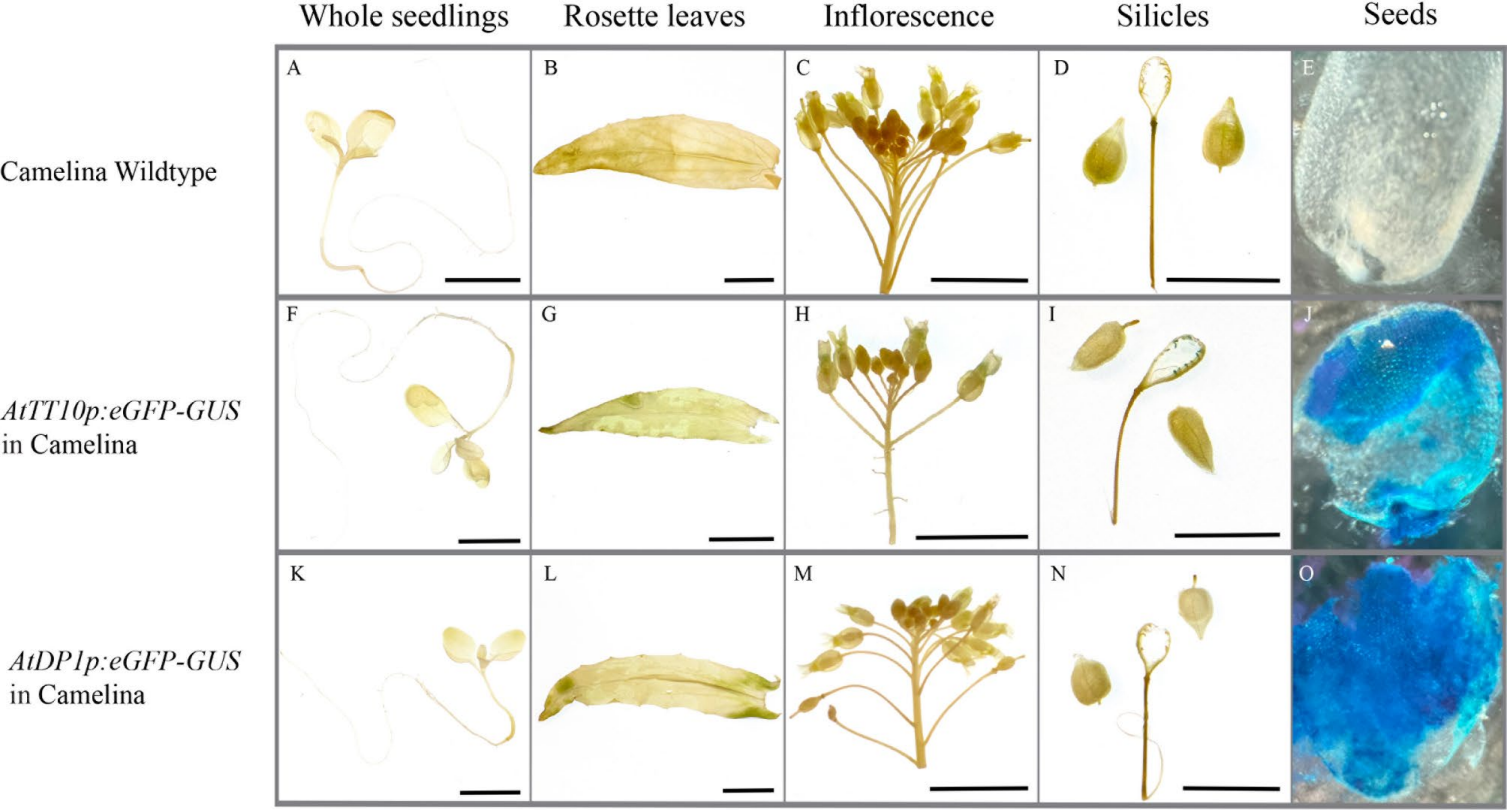
GUS signals showing *AtTT10* and *AtDP1* promoters activities in transgenic camelina different tissues. One week old whole seedlings (**A, F** and **K**), rosette leaves of mature plants (**B, G** and **L**), inflorescence (**C, H** and **M**), silicles (hand peeled open and removed seeds) (**D, I** and **N**) and seeds (cut open along the longitudinal direction with the cutting sides facing down) (**E, J** and **O**) of wildtype (**A-E**), transgenic line carrying *AtTT10p:eGFP-GUS* (**F-J**) and transgenic line carrying *AtDP1p:eGFP-GUS* (**K-O**) were treated with GUS staining. Scale bars = 1 cm

We performed GUS staining in different tissues of the transgenic pennycress and camelina, including one week old whole seedlings, rosette leaves from mature plants, inflorescence, green silicles, and seeds. Non-transgenic wild-type plants were used as a negative control. In pennycress, among the tissues examined, GUS staining was only observed at the seed surface for *AtDP1p:eGFP-GUS*, whereas the GUS signal has a wider distribution for *AtTT10p:eGFP-GUS* (Fig. 1). Besides the strong GUS signals in seeds, transgenic pennycress carrying *AtTT10p:eGFP-GUS* showed low levels of GUS staining in cotyledons, flowers, and the septa of silicles. In camelina, no GUS signal was found in tissues other than the seeds except the presence of trace amount of staining in the funiculi on the septa of silicles that directly connect with seeds for *AtTT10p:eGFP-GUS* (Fig. 2).

### *AtTT10* and *AtDP1* promoters show different expression profiles during seed development in pennycress and camelina

**Fig. 3:**
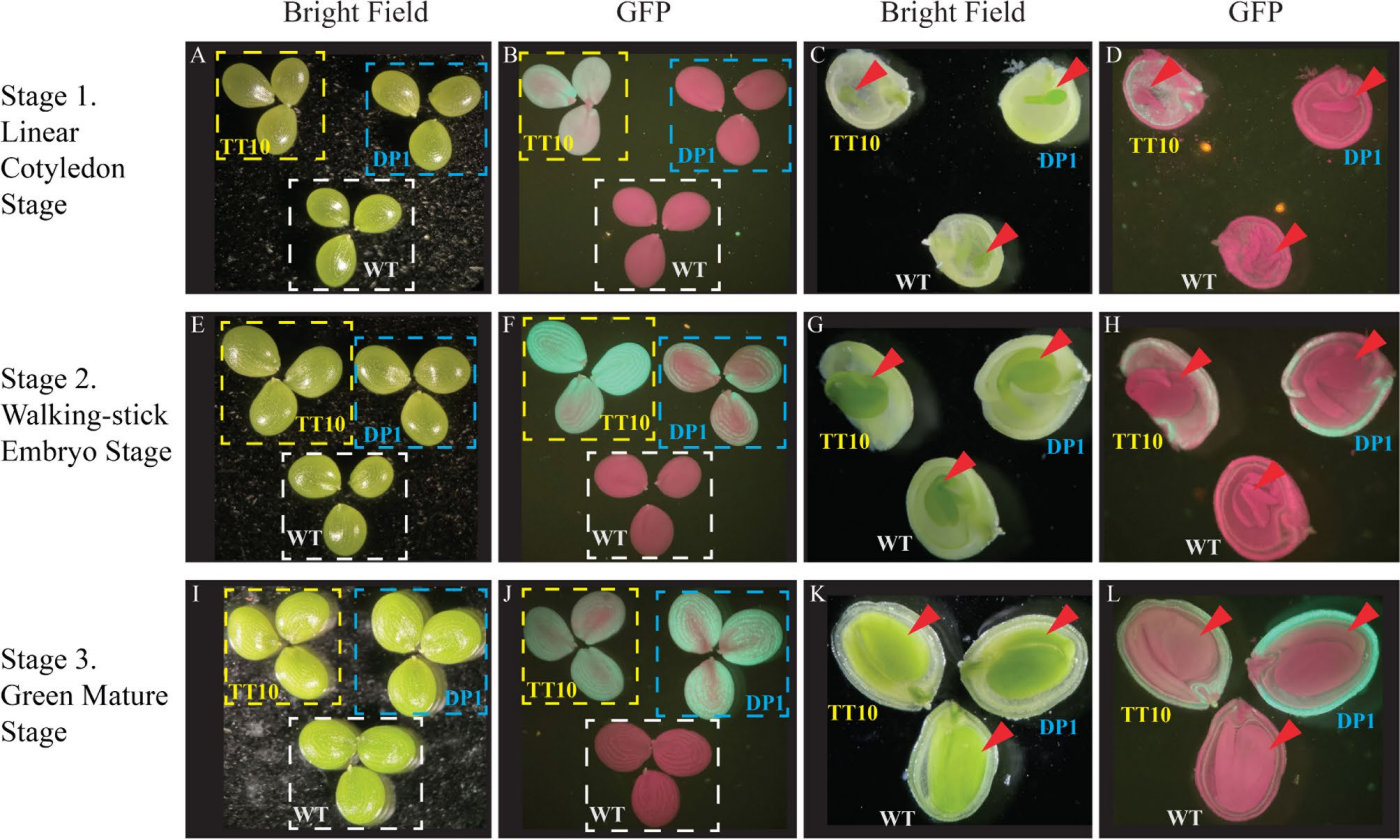
GFP signals showing *AtTT10* and *AtDP1* promoter activities in transgenic pennycress seeds. (**A**) Three seeds of pennycress WT (‘MN108’), transgenic pennycress with *AtTT10p:eGFP-GUS* (labeled as TT10 in short), and transgenic pennycress with *AtDP1p:eGFP-GUS* (labeled as DP1 in short), were arranged into a single view field of a dissecting microscope and observed under bright field. Panel B, E, F, I and J were organized the same way. (**B**) The same samples in panel A were observed under the GFP florescent channel. (**C**) One seed from each of the three genotypes shown in panel A and B was cut along the longitudinal direction with the cutting sides facing up, and observed using a dissecting microscope under bright field. Panel D, G, H, K and L were organized the same way. Arrows indicate the embryos in each dissected seed. (**D**) The same samples in panel C were observed under the GFP florescent channel. (**A-D**) Seeds at linear cotyledon development stage. (**E-H**) Seeds at the walking-stick embryo development stage. (**I-L**) Seeds at the green mature development stage. Field area of panel A, B, E, F, I and J are the same; field area of panel C, D, G, H, K and L are the same

**Fig. 4:**
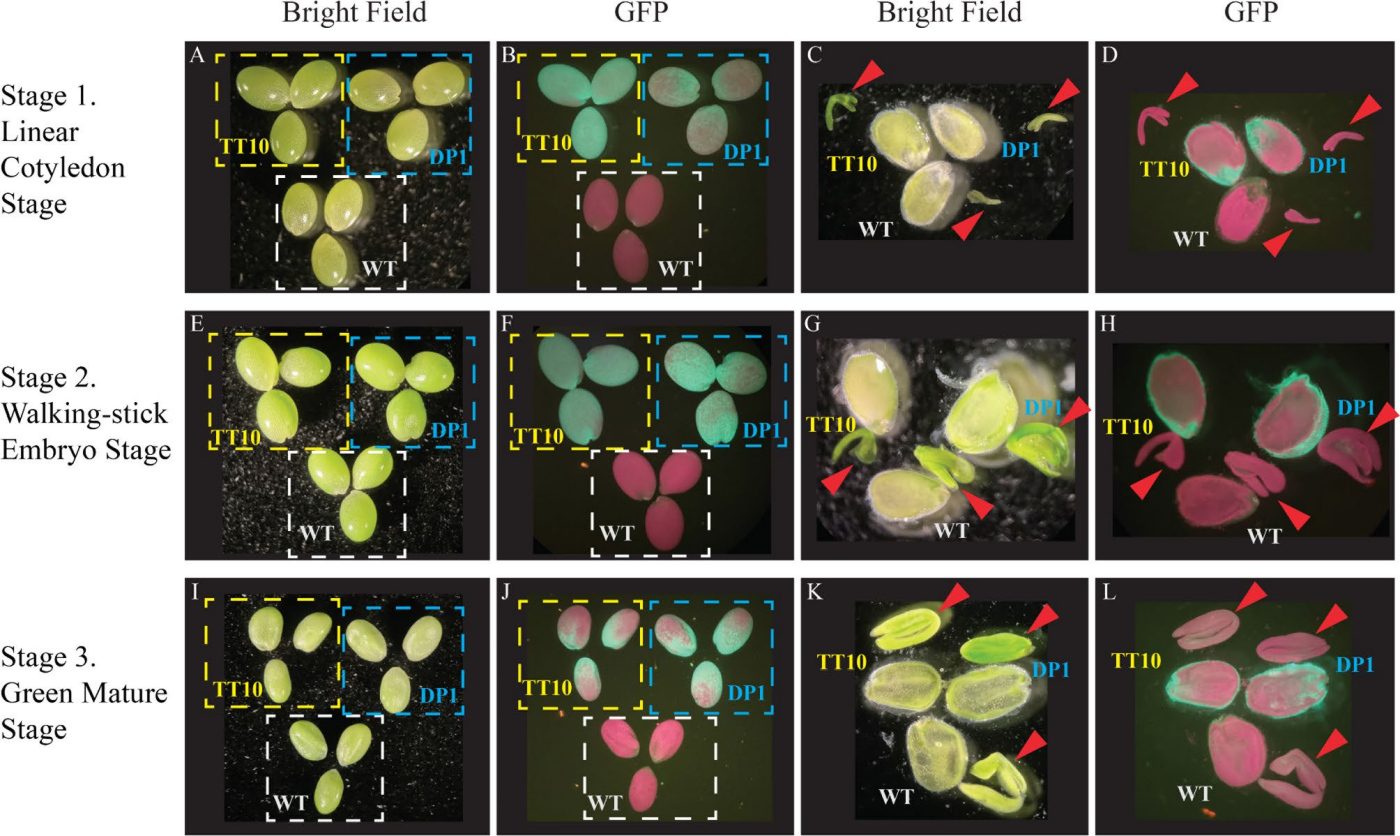
GFP signals showing *AtTT10* and *AtDP1* promoter activities in transgenic camelina seeds. (**A**) Three seeds of camelina WT (‘Suneson’), transgenic camelina with *AtTT10p:eGFP-GUS* (labeled as TT10 in short), and transgenic camelina with *AtDP1p:eGFP-GUS* (labeled as DP1 in short), were arranged into a single view field of a dissecting microscope and observed under bright field. Panel B, E, F, I and J were organized the same way. (**B**) The same samples in panel A were observed under the GFP florescent channel. (**C**) One seed from each of the three genotypes shown in panel A and B was cut along the longitudinal direction with the cutting sides facing up, and observed using a dissecting microscope under bright field. Panel D, G, H, K and L were organized the same way. Arrows indicate the embryos in each dissected seed, the embryos were placed next to the seed coat tissue. (**D**) The same samples in panel C were observed under the GFP florescent channel. (**A-D**) Seeds at linear cotyledon development stage. (**E-H**) Seeds at the walking-stick embryo development stage. (**I-L**) Seeds at the green mature development stage. Field area of panel A, B, E, F, I and J are the same; field area of panel C, D, G, H, K and L are the same

To better understand the activities of the two promoters in pennycress and camelina seed coat during seed development, we monitored the GFP signal in the seed coat during five different stages: the heart stage, linear cotyledon stage, walking-stick embryo stage, green mature stage, and the brown mature stage. Developmental stages are defined by embryo morphology based on Arabidopsis research [19, 21]. GFP signals were not detected from seeds at the heart stage and the brown mature stage, and thus we focused on the middle three stages for more careful characterization. In addition to imaging the intact seed, we also cut open a seed to gain additional information on the location of the GFP signal within the seed. Consistent with the inference from whole seed GUS staining data, *AtTT10* and *AtDP1* promoters drive gene expression in the seed coat in both pennycress (Fig. 3) and camelina (Fig. 4).

In pennycress, *AtTT10* promoter drove eGFP expression in the seed coat at all three stages, with the walking-stick embryo stage showing the highest GFP signal intensity among the three stages observed, whereas the *AtDP1* promoter driving eGFP expression started during the walking-stick embryo stage and increased at the green mature stage (Fig. 3). In camelina, both *AtTT10* and *AtDP1* promoters drove eGFP expression in the seed coat during all three developmental stages; however, the strongest expression was at the linear cotyledon stage for *AtTT10* promoter and the walking-stick stage for *AtDP1* promoter (Fig. 4).

### *AtTT10* and *AtDP1* promoters are mainly active in outer integument of seed coat

**Fig. 5:**
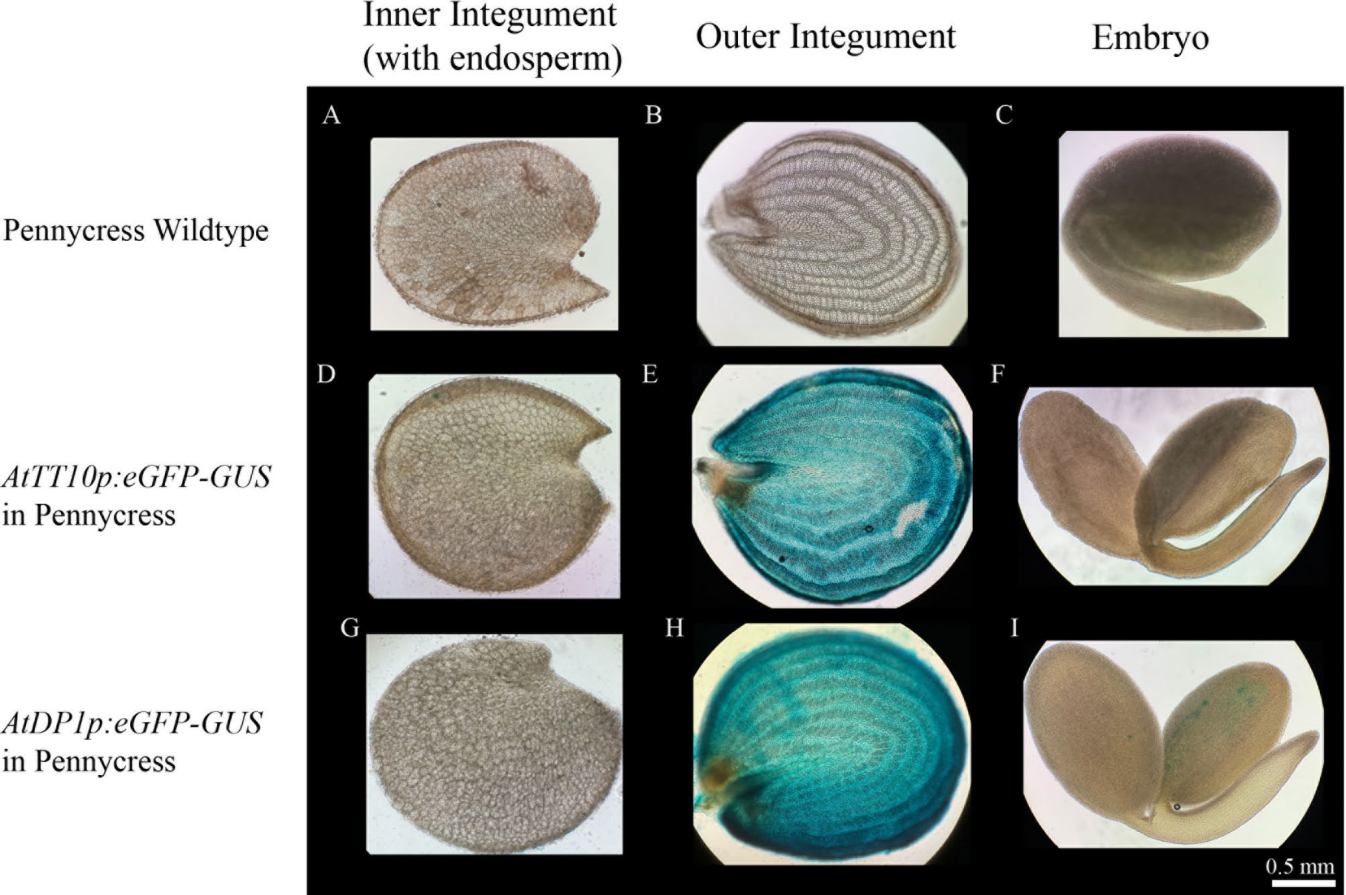
GUS signals showing *AtTT10* and *AtDP1* promoter activities in transgenic pennycress seeds. Green mature stage of seeds of wildtype (**A-C**), transgenic line carrying *AtTT10p:eGFP-GUS* (**D-F**) and transgenic line carrying *AtDP1p:eGFP-GUS* (**G-I**) were cut along the longitudinal direction, and then manually separated into three parts, namely inner integument layers with peripheral endosperm tissue (**A, D** and **G**), outer integument layers (**B, E** and **H**), and embryos (**C, F** and **I**), and then treated with GUS staining. Scale bar = 0.5 mm

**Fig. 6:**
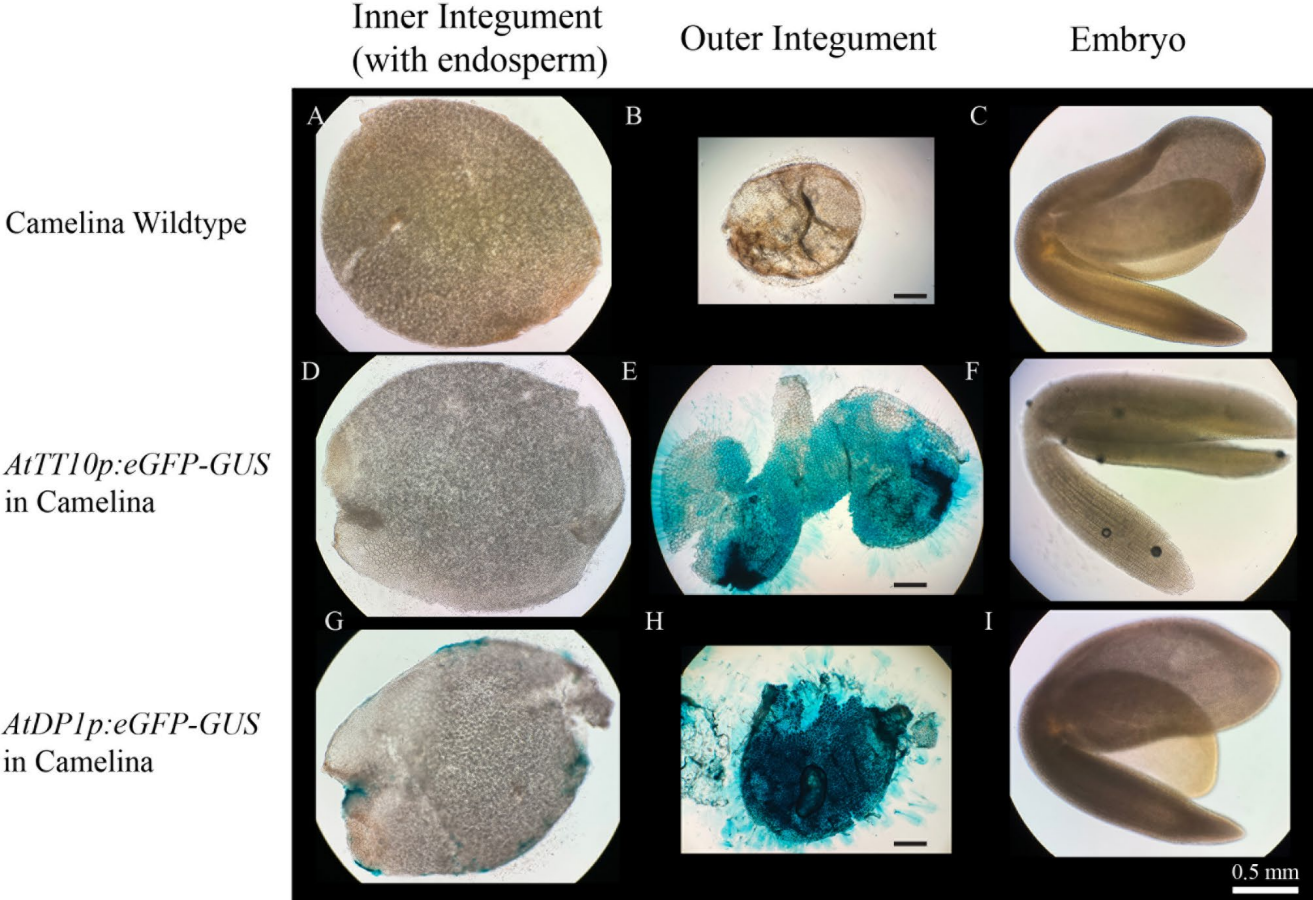
GUS signals showing *AtTT10* and *AtDP1* promoter activities in transgenic camelina seeds. Green mature stage of seeds of wildtype (**A-C**), transgenic line carrying *AtTT10p:eGFP-GUS* (**D-F**) and transgenic line carrying *AtDP1p:eGFP-GUS* (**G-I**) were cut along the longitudinal direction, and then manually separated into three parts, namely inner integument layers with peripheral endosperm tissue (**A, D** and **G**), outer integument layers (**B, E** and **H**), and embryos (**C, F** and **I**), and then treated with GUS staining. Scale bar = 0.5 mm

As revealed by the GFP data, *AtTT10* and *AtDP1* promoters seemed to drive gene expression mainly in the outer integument of the seed coat in pennycress and camelina. To further investigate in which seed coat layer the two promoters are active, we separated different parts of the seed and examined them with GUS staining. Seeds of green mature stage were hand dissected into three parts: the outer integument, the inner integument with attached peripheral endosperm, and the embryo. These parts were collected separately and stained for GUS expression. In transgenic pennycress and camelina that carry either *AtTT10p:eGFP-GUS* or *AtDP1p:eGFP-GUS*, intense blue color was observed only in the outer integument while the inner integument and the embryo were mostly free from GUS staining (Figs. 5 and 6). This expression pattern generally holds true for seeds at other stages examined in this study, as evident from the GFP reporter signals (Figs. 3 and 4). The only exception was noticed at the early cotyledon embryo stage in transgenic pennycress carrying *AtTT10p:eGFP-GUS* where the GFP signal was seen in the inner integument instead (Fig. 3D and Supplemental Figure S3).

## Discussion

### Both *AtTT10* and *AtDP1* drive gene expression predominantly in seed coat in pennycress and camelina

Seed coat specific promoters are important biotechnology tools for engineering seed traits in crops. Testing the promoter activity of well-characterized Arabidopsis genes in *Brassicaceae* oilseed crop species is an attractive approach which bypasses the requirement of comprehensive transcriptome analysis in the target species to discover candidate promoters. Several Arabidopsis seed coat specific gene promoters including *TT10*, *DP1*, *GILT* [29], *BAN* [27], and *δVPE* [28] have been tested in canola [30, 33–35]. In this study, we tested the activity of Arabidopsis *TT10* and *DP1* promoters in two emerging biofuel oilseed crops, pennycress and camelina. In general, both *AtTT10* and *AtDP1* promoters show strong activity in the seed coat and little to no activity in other tissues examined. This seed coat specific expression pattern observed in pennycress and camelina is similar to the activities of the *AtTT10* and *AtDP1* promoters in Arabidopsis [23, 30, 41] and canola [30, 33].

### *AtTT10* promoter behaves differently in pennycress and camelina

Despite the overall similarity in the promoter activity, there were notable differences in the expression pattern of *AtTT10* promoter between pennycress and camelina. In pennycress, *AtTT10* promoter activity was detected in the inner integument of seed coat at the linear cotyledon stage but in the outer integument instead during later stages (Fig. 3D and Supplemental Figure S3), which is consistent with the observation in Arabidopsis [23]. In contrast, *AtTT10* promoter activity was only detected in the outer integument in camelina (Fig. 4D), which is similar to the observation in canola [33].

*AtTT10* was reported to encode a laccase that functions in oxidative polymerization of flavonoids and monolignols in the seed coat. Arabidopsis *tt10* mutants showed a delay in seed coat browning, accumulates less lignin, and has more soluble proanthocyanidins in seeds than wild type [23, 24, 42]. It is possible that *TT10* promoter activity in inner integument and outer integument is responsible for producing the enzyme required for polymerization of proanthocyanidins and lignin, respectively. The lack of activity in inner integument in camelina and canola suggests that some *cis* elements in *AtTT10* promoter or their interaction with *trans* factors may not be conserved between Arabidopsis and these two species.

Apart from in the seed coat, very low levels of GUS signals were detected in the seedlings and flowers in pennycress carrying *AtTT10p:eGFP-GUS* (Fig. 1F and 1H). This expression pattern is consistent with the report demonstrating that Arabidopsis showed low levels of *AtTT10* expressions in stems, seedlings, and flowers [23]. However, no *AtTT10* promoter activities were seen in camelina seedlings and flowers (Fig. 2F and 2H), similar to what was observed in canola [33].

These results suggest that the activity of a promoter may differ even in closely related species. Such species-dependent expression pattern differences have also been observed for other genes. For example, Arabidopsis *δVPE* promoter showed activities mainly in the inner integument of Arabidopsis seed coat [28], but in transgenic canola, it was mainly active in outer integument [34]. Therefore, careful examination of exogenous promoter activities in target species remains necessary, even when species are closely related.

### *AtTT10* promoter has a broader expression activity than *AtDP1* promoter

While both *AtTT10* and *AtDP1* promoters showed major activity in the outer integument of seed coat in both pennycress and camelina, these two promoters have slightly different expression profiles. In both species, *AtDP1* promoter’s activity is more specific to the seed coat than the *AtTT10* promoter (Figs. 1 and 2). We also observed differences in the growth stage at which these promoters exhibit peak activity. In pennycress, the *AtTT10* promoter signal was strongest in the walking-stick stage and remained high during the green mature stage; while the *AtDP1* promoter showed the highest activity during the green mature stage (Fig. 3). In camelina, the *AtTT10* promoter activity appeared strongest at the linear cotyledon stage and became increasingly weaker during the later stages, whereas *AtDP1*-driven GFP signal peaked at the walking-stick stage (Fig. 4).

### Potential application of *AtTT10* and *AtDP1* promoters to improve pennycress and camelina

An increasing amount of evidence suggests that seed coat pigmentation and lignification is negatively associated with seed oil production and meal quality [43–48]. Flavonoid pigments and lignin are both derived from the phenylpropanoid pathway, which produces a wide range of products in different plant tissues serving critical functions [49, 50]. Modifying phenolic traits for oilseed improvement thus requires manipulating genes and pathways in a seed coat tissue-specific manner to avoid the negative effects associated with broad perturbation of phenylpropanoid production and plant growth.

In addition to removing negative seed coat qualities, utilizing seed coat specific promoters may offer a promising route to augmenting the pennycress and camelina seed value. Press meal for feeding livestock, a byproduct of seed oil extraction, has been an important target trait during pennycress and camelina domestication [51–53]. Specifically, the seed coat tissue specific expression of bioactive compounds can substantially improve meal quality and value. An attractive candidate of gain-of-function strategy of this kind is to employ seed coat promoters to drive expression of *QsuB* (3-dehydroshikimate dehydratase). This application not only induced biosynthesis and accumulation of protocatechuate (PCA), a phenolic bioproduct with recognized health benefits, but also reduces lignin production [54, 55].

Another attractive area is in manipulating the production of seed mucilage, particularly in camelina. For basic research, seed mucilage offers a unique window to study plant polysaccharide biosynthesis [56]. In the meantime, mucilage production has garnered attention in the food and chemical industry due to its utility as a plant-derived polymer [57]. The application of seed coat specific promoters enables precise engineering of mucilage characteristics, mitigating the risk of disturbing plant normal growth.

Our analyses demonstrate that both *AtTT10* and *AtDP1* promoters are suitable for driving gene expression in seed coat in pennycress and camelina. The observed differences in the temporal and spatial expression patterns between these two promoters add nuance to our ability of engineering seed coat traits in the two emerging non-food oilseed crops. The choice of promoter depends on the specific target application and species. For example, *AtDP1* promoter is notably more active during the later stages of seed coat development in pennycress, at the green mature stage, marking it more suitable for applications aimed at this particular phase of pennycress seed development.

### Electronic supplementary material

Below is the link to the electronic supplementary material.

**Supplemental Table S1**. Primers used in this study. **Supplemental Figure S1**. Plasmid maps of the two transgenic constructs *AtTT10p:eGFP-GUS* and *AtDP1p:eGFP-GUS*. **Supplemental Figure S2**. Transgenic pennycress (left) and camelina (right) seedlings selection on MS media plates containing hygromycin for one week. Arrows indicate seedlings showing resistance to hygromycin. **Supplemental Figure S3**. A higher magnification observation of *AtTT10p:eGFP-GUS* in transgenic pennycress at the linear cotyledon stage under the GFP channel (same specimen as shown in Figure 3D). The seed was cut open along the longitudinal axis with the inside of the seed facing up. Scale bar = 0.2 mm.

## Data Availability

The datasets generated and analyzed during this work are available from the corresponding author on reasonable request.
