# Supplementary material for: Two Arabidopsis promoters drive seed-coat specific gene expression in pennycress and camelina
Source: Plant Methods. 2023 Dec 6;19:140. doi: 10.1186/s13007-023-01114-x (PMC10699083; doi:10.1186/s13007-023-01114-x)

**Supplementary Information**

**Article title:** Two Arabidopsis promoters drive seed-coat specific gene expression in pennycress and camelina

**Journal name:** Plant Methods

**Author names:** Xin Li, Victoria Yell and Xu Li.

**Affiliation:** Department of Plant and Microbial Biology, North Carolina State University, Raleigh, NC 27695; and Plants for Human Health Institute, North Carolina State University, Kannapolis, NC 28081, USA.

**e-mail address of the corresponding author:** Xu Li (sirius_li@ncsu.edu).

**Supplemental Table S1**. Primers used in this study.

| Primers | Sequence (5’-3’) | Purpose |
| --- | --- | --- |
| oXL1968 | GGTAATAAATAGGAAAGTCTGGTAACACACTGATTTTGCTTGGAATG | Amplify *AtTT10* promoter |
| oXL1969 | GCCCTTGCTCACCATTTTGGAAGAGTTTTAGTAAATTAATTACCCTG | Amplify *AtTT10* promoter |
| oXL1970 | GGTAATAAATAGGAAAGTCTGGTAGCTTTTCTGGGAAGCTCGTT | Amplify *AtDP1* promoter |
| oXL1971 | GCCCTTGCTCACCATTGTTAGAGTGTTAAGTAGAAATGAGTAGTATTG | Amplify *AtDP1* promoter |
| oXL2124 | GCGTCCATGTCAGCAATTCA | *AtTT10p:eGFP-GUS* construct genotyping |
| oXL2125 | CCGGACGAAATGTTCCCCTA | *AtTT10p:eGFP-GUS* construct genotyping |
| oXL2122 | GTATCAGTGTGCATGGCTGG | *AtDP1p:eGFP-GUS* construct genotyping |
| oXL2123 | GTCTGCCACGGTCTAGATGA | *AtDP1p:eGFP-GUS* construct genotyping |

**Supplemental Figure S1**. Plasmid maps of the two transgenic constructs *AtTT10p:eGFP-GUS* and *AtDP1p:eGFP-GUS*.


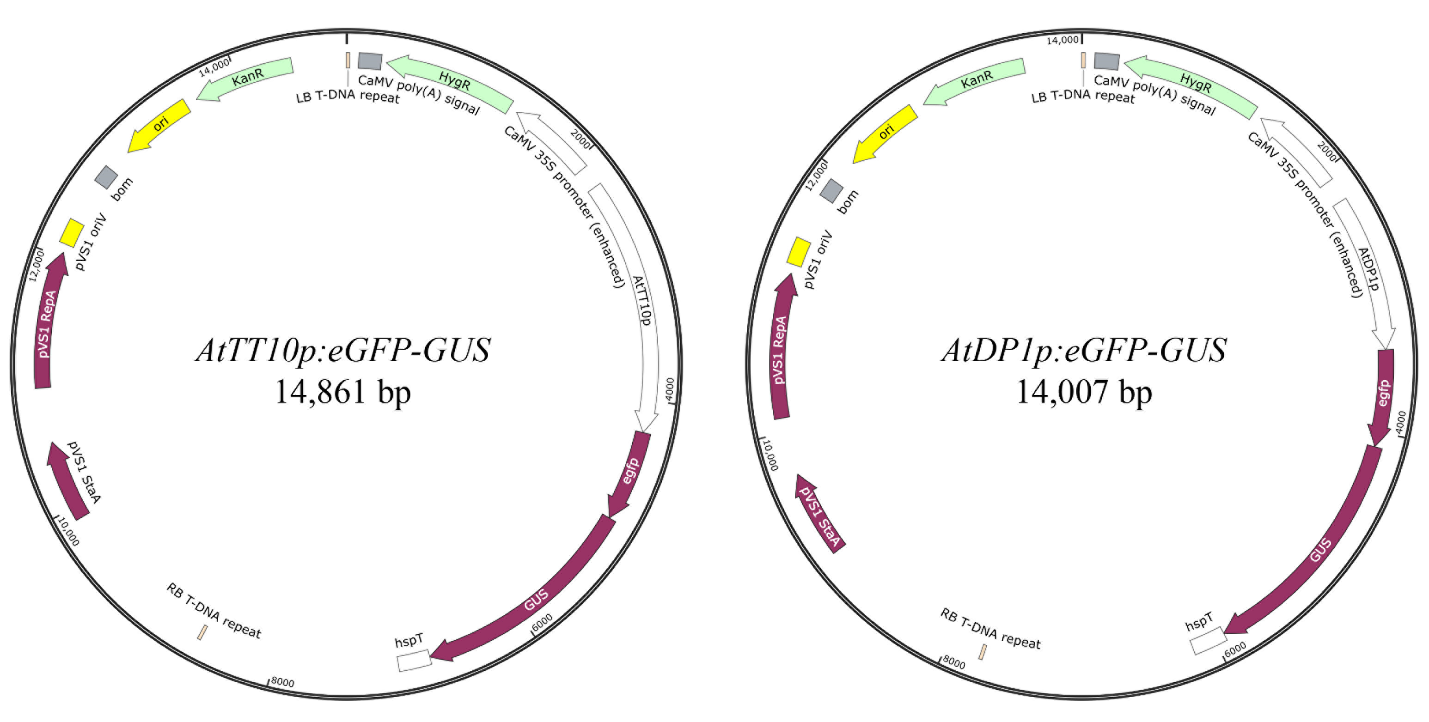


**Supplemental Figure S2**. Transgenic pennycress (left) and camelina (right) seedlings selection on MS media plates containing hygromycin for one week. Arrows indicate seedlings showing resistance to hygromycin.


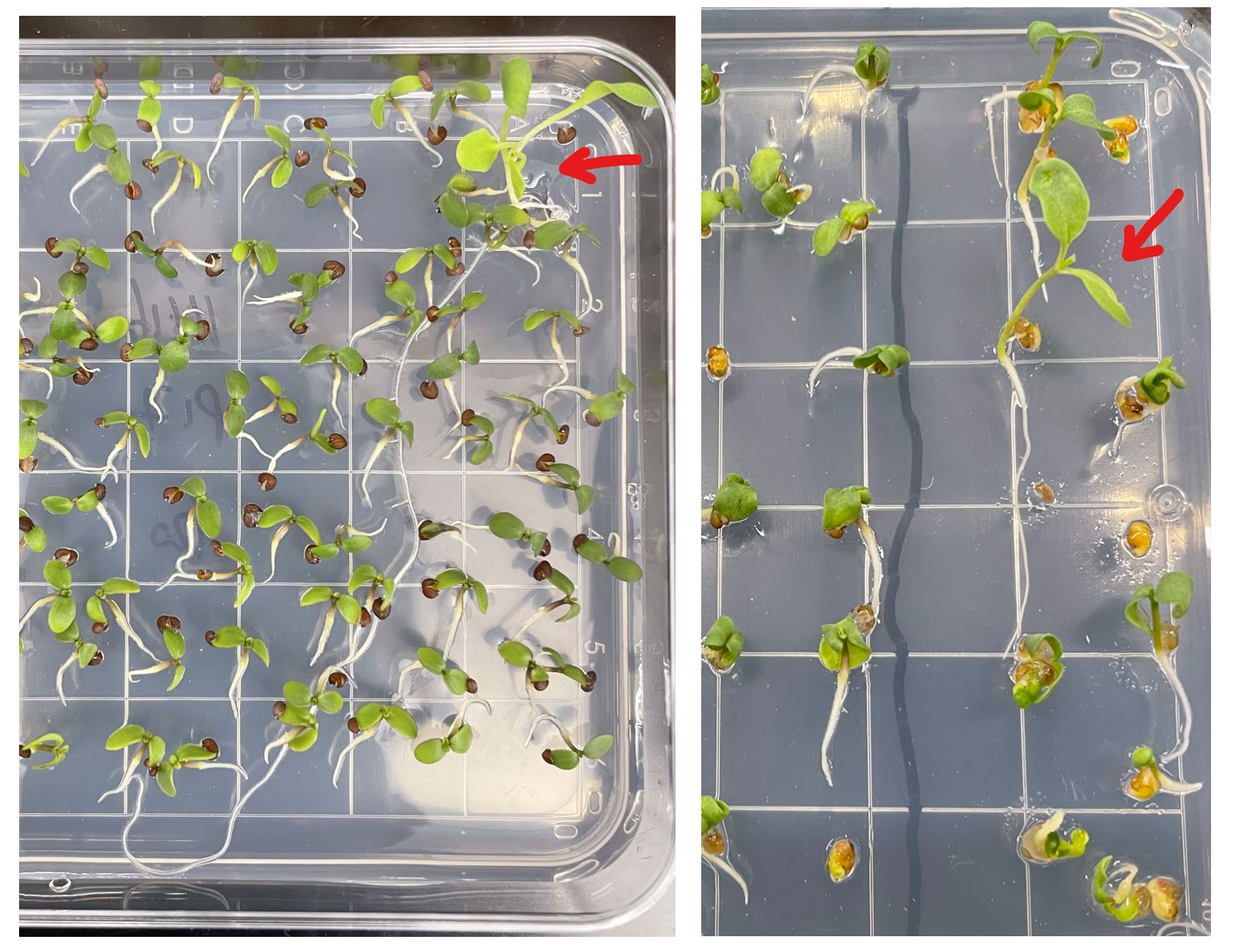


**Supplemental Figure S3.** A higher magnification observation of *AtTT10p:eGFP-GUS* in transgenic pennycress at the linear cotyledon stage under the GFP channel (same specimen as shown in Figure 3D). The seed was cut open along the longitudinal axis with the inside of the seed facing up. Scale bar = 0.2 mm.


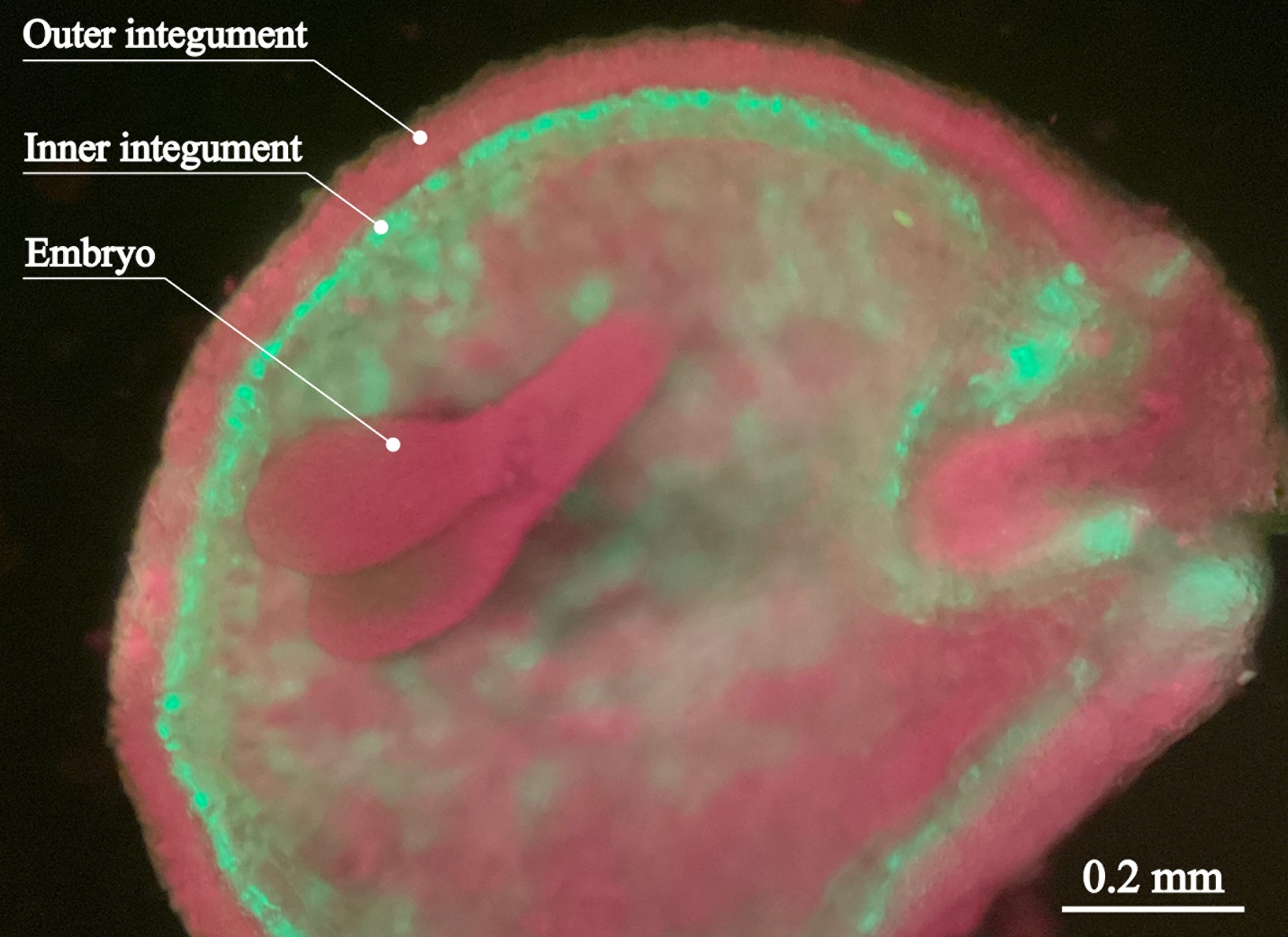

Supplement: Supplementary file 1 — Supplemental Table S1. Primers used in this study. Supplemental Figure S1. Plasmid maps of the two transgenic constructs AtTT10p:eGFP-GUS and AtDP1p:eGFP-GUS. Supplemental Figure S2. Transgenic pennycress (left) and camelina (right) seedlings selection on MS media plates containing hygromycin for one week. Arrows indicate seedlings showing resistance to hygromycin. Supplemental Figure S3. A higher magnification observation of AtTT10p:eGFP-GUS in transgenic pennycress at the linear cotyledon stage under the GFP channel (same specimen as shown in Figure 3D). The seed was cut open along the longitudinal axis with the inside of the seed facing up. Scale bar = 0.2 mm. [file 13007_2023_1114_MOESM1_ESM.docx]
